# Structural analysis of group II chitinase (ChtII) catalysis completes the puzzle of chitin hydrolysis in insects

**DOI:** 10.1074/jbc.RA117.000119

**Published:** 2018-01-09

**Authors:** Wei Chen, Mingbo Qu, Yong Zhou, Qing Yang

**Affiliations:** From the ‡ State Key Laboratory of Fine Chemical Engineering, School of Life Science and Biotechnology and School of Software, Dalian University of Technology, Dalian 116024, China and; the § Institute of Plant Protection, Chinese Academy of Agricultural Sciences, Beijing 100193, China

**Keywords:** chitin, chitinase, crystal structure, insect, enzyme catalysis, enzyme structure, chitin degradation, Ostrinia furnacalis, Serratia marcescens

## Abstract

Chitin is a linear homopolymer of *N*-acetyl-β-d-glucosamines and a major structural component of insect cuticles. Chitin hydrolysis involves glycoside hydrolase family 18 (GH18) chitinases. In insects, chitin hydrolysis is essential for periodic shedding of the old cuticle ecdysis and proceeds via a pathway different from that in the well studied bacterial chitinolytic system. Group II chitinase (ChtII) is a widespread chitinolytic enzyme in insects and contains the greatest number of catalytic domains and chitin-binding domains among chitinases. In Lepidopterans, ChtII and two other chitinases, ChtI and Chi-h, are essential for chitin hydrolysis. Although ChtI and Chi-h have been well studied, the role of ChtII remains elusive. Here, we investigated the structure and enzymology of *Of*ChtII, a ChtII derived from the insect pest *Ostrinia furnacalis*. We present the crystal structures of two catalytically active domains of *Of*ChtII, *Of*ChtII-C1 and *Of*ChtII-C2, both in unliganded form and complexed with chitooligosaccharide substrates. We found that *Of*ChtII-C1 and *Of*ChtII-C2 both possess long, deep substrate-binding clefts with endochitinase activities. *Of*ChtII exhibited structural characteristics within the substrate-binding cleft similar to those in *Of*Chi-h and *Of*ChtI. However, *Of*ChtII lacked structural elements favoring substrate binding beyond the active sites, including an extra wall structure present in *Of*Chi-h. Nevertheless, the numerous domains in *Of*ChtII may compensate for this difference; a truncation containing one catalytic domain and three chitin-binding modules (*Of*ChtII-B4C1) displayed activity toward insoluble polymeric substrates that was higher than those of *Of*Chi-h and *Of*ChtI. Our observations provide the last piece of the puzzle of chitin hydrolysis in insects.

## Introduction

Chitin is a linear homopolymer of *N*-acetyl-β-d-glucosamines. Hydrolysis of chitin, which is a major structural component of insect cuticles, allows insects to overcome the growth limitation imposed by the old cuticle during growth and development (1). For bacteria, hydrolysis of chitin fulfills their nutrient demands for carbon and nitrogen sources and plays a role in bacterial pathogenesis as well (2).

The glycoside hydrolase family (GH)^3^ 18 chitinases (EC 3.2.1.14) are essential enzymes for chitin hydrolysis (3, 4). All GH18 chitinases adopt the substrate-assisted mechanisms; the formation of a covalent oxazolinium ion intermediate requires distortion of the −1 sugar toward a boat conformation, which enables the C2-acetamido group to act as the catalytic nucleophile. In the extensively studied microbial system of *Serratia marcescens*, the three chitinases *Sm*ChiA, *Sm*ChiB, and *Sm*ChiC are known to cleave chitin chains from the reducing end, from the non-reducing end, and at random internal sites, respectively (5). The different architectures of the substrate-binding clefts of *Sm*ChiA, *Sm*ChiB, and *Sm*ChiC confer different catalytic properties to these enzymes, which work synergistically in chitin hydrolysis (5–9).

The pattern of chitin hydrolysis in insects appears to differ from that of bacteria. In the lepidopteran pest *Ostrinia furnacalis*, three chitinases *Of*ChtI, *Of*ChtII, and *Of*Chi-h are essential for hydrolysis. The crystal structures of *Of*ChtI and *Of*Chi-h have been determined. *Of*ChtI contains a long open groove-like substrate-binding cleft, exhibiting similarity to the exo-acting *Sm*ChiB, and shares structural features with the endo-acting chitinase *Sm*ChiC (10). *Of*Chi-h contains a substrate-binding cleft with the structural characteristics of the exo-acting chitinase *Sm*ChiA (11). Together, *Of*ChtI and *Of*Chi-h are functionally equivalent to the combination of *Sm*ChiA, *Sm*ChiB, and *Sm*ChiC. Indeed, synergism between *Of*ChtI and *Of*Chi-h has been observed *in vitro* (11). Thus, the role of ChtII in the biodegradation of chitin is unclear.

ChtII is a GH18 chitinase and possesses the greatest number of catalytic domains and chitin-binding domains in this family. Insect ChtII enzymes generally have 4–5 catalytic domains and 4–7 chitin-binding domains. In all insects studied so far, only one gene encodes ChtII, and it is expressed throughout all molting stages. Sequences of the ChtII genes have been determined for most insect orders, including Lepidoptera, Coleoptera, Diptera, Hemiptera, Hymenoptera, Orthoptera, and Phthiraptera (12–22). In addition, there is mounting evidence showing that ChtII is essential to successful molting. Specific knockdown of ChtII transcripts in the coleopteran *Tribolium castaneum* prevented larval–, larval–pupal, and pupal–adult molting and egg hatching (23). Feeding the dsRNA of ChtII to larvae of the lepidopteran *O. furnacalis* resulted in death caused by defective molting (24). For the lepidopteran *Chilo suppressalis*, ChtII dsRNA–treated last-instar larvae were arrested at the stage of pupa and died eventually (21). Although a physiological role for ChtII during molting is certain, biochemical characterization of ChtII is lacking.

Here we report the catalytic properties and the crystal structures of the catalytic domains of *Of*ChtII, the ChtII derived from the Asian corn borer *O. furnacalis*. This work provides insights into the catalysis of ChtII, shedding light on synergism among insect chitinases. Because ChtII is essential for molting, this structural information offers a possibility for development of novel agrochemicals for control of insect pests.

## Results

### Sequence of OfChtII

**Figure 1.**
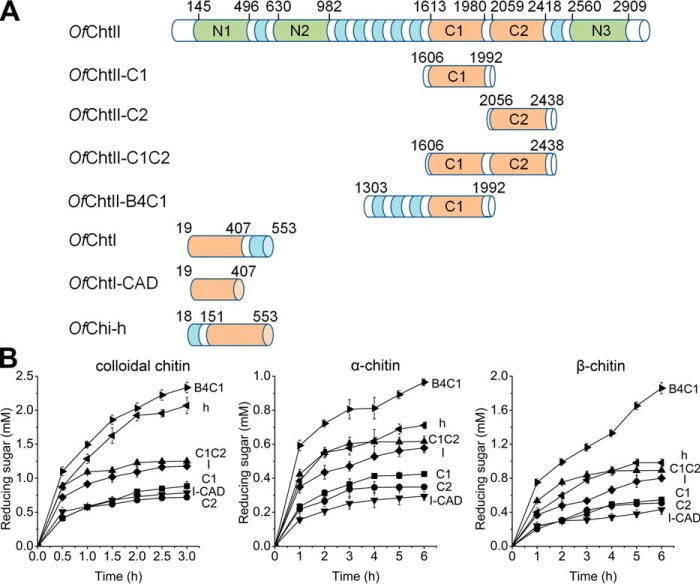
**Domain architecture and hydrolytic activities of chitinases.** *A*, domain architecture of the enzyme used in this study. Active catalytic domains, inactive catalytic domains, chitin-binding domains, and linker regions are highlighted in *orange*, *green*, *blue*, and *white*, respectively. *B*, the hydrolytic activities of chitinases toward different substrates. Products were measured during degradation of polymer chitin substrates, colloidal chitin (*left panel*), α-chitin (*middle panel*), and β-chitin (*right panel*). The reaction mixtures contained identical amounts of enzyme and substrate. The results are the average of three independent repeats, with the standard deviations indicated.

Based on the conserved sequence and domain similarities among group II insect chitinases, a 9077-nucleotide complementary DNA (cDNA) containing an open reading frame encoding a protein containing 2929 amino acids (*Of*ChtII, GenBank^TM^ accession number MF034108) was obtained from the insect pest, *O. furnacalis*. The first 17 amino acid residues were predicted to be the signal peptide. Analyzing the domain structure of the predicted protein with the SMART tool (25) revealed that *Of*ChtII contains five GH18 domains and seven CBM14-type chitin-binding modules (CBMs) (Fig. 1*A*). The number and order of GH18 domains and CBMs were precisely similar to those in other lepidopterans according to multiple sequence alignment (Fig. S1). All GH18 domains contained four highly conserved motifs: K*XXXXX*GGW, FDG*X*DLDWEYP, M*X*YD*XX*G, and G*XXX*W*XX*D*X*DD (where *X* represents a non-specified amino acid; Fig. S2) (26). The glutamate in the second conserved motif, FDG*X*DLDWEYP, was shown to be the proton donor required for cleavage of the glycosidic bond. Because proteins with substitutions in this residue (Glu → Asn or Glu → Gln) have been shown to have very little or no enzymatic activity (27), the GH18 domains N1, N2, and N3 in which the catalytically critical Glu residue are mutated to Val, Asn, or Gln, respectively, are presumed to be inactive. The other two GH18 domains (C1 and C2) are predicted to be catalytically active (CAD).

### Enzymatic activities of OfChtII

To gain information on the catalytic features of *Of*ChtII, the two catalytically active domains (C1 and C2) were cloned and expressed separately or together. The enzymatic activities were measured using different substrates including 4-methylumbelliferyl β-d-*N*,*N′-*diacetylchitobioside hydrate (MU-(GlcNAc)_2_),α-chitin, β-chitin, and colloidal chitin (Table 1 and Fig. 1*B*). *Of*ChtII-C1 and *Of*ChtII-C2 showed similar activities toward all of the substrates tested, and the activity of the construct with both catalytic domains (*Of*ChtII-C1C2) was nearly equal to the sum of two single domain activities, suggesting that there is no synergistic effect between *Of*ChtII-C1 and *Of*ChtII-C2.

**Table 1:** **Apparent steady-state kinetic parameters of different chitinases for MU-(GlcNAc)_2_** The results are the averages of three independent repeats with the standard deviations indicated.

Enzyme	*K_m_*	*s*_cat_	*k*_cat_/*K_m_*
	μ*m*	*min*^−*1*^	*min*^−*1*^ μ*m*^−*1*^
*Of*ChtII-C1	15.52 ± 0.98	32.99 ± 0.65	2.13
*Of*ChtII-C2	11.93 ± 0.50	12.22 ± 0.15	1.02
*Of*ChtII-C1C2	15.27 ± 0.67	46.69 ± 2.76	3.06
*Of*ChtII-B4C1	16.37 ± 0.85	28.54 ± 0.83	1.74
*Of*ChtI-CAD	1.56 ± 0.07	69.84 ± 4.92	44.77
*Of*ChtI	2.23 ± 0.06	107.26 ± 2.31	48.05
*Of*Chi-h	7.32 ± 0.35	188.27 ± 5.31	25.72

Compared with *Of*ChtI, *OfChtI*-CAD (a truncation containing only the catalytic domain of *Of*ChtI), *Of*Chi-h, *Of*ChtII-C1, and *Of*ChtII-C2 exhibit substantially lower activities toward the small molecule substrate MU-(GlcNAc)_2_ (Table 1). *Of*ChtII-B4C1, which includes three adjacent CBMs followed by a catalytically active CAD (C1), showed similar activity with *Of*ChtII-C1.

For polymeric substrates, there were no significant differences among *Of*ChtII-C1, *Of*ChtII-C2, and *Of*ChtI-CAD. *Of*Chi-h and *Of*ChtI showed higher activities than enzymes containing a single catalytic domain, in that they retained the chitin-binding domains (fibronectin III type chitin-binding domain in *Of*Chi-h and CBM-14 type chitin-binding domain in *Of*ChtI), suggesting the importance of the chitin-binding module in hydrolysis of crystalline chitin. Consistent with this observation, *Of*ChtII-B4C1 showed the highest hydrolytic activity. Interestingly, by combining two CADs, the hydrolytic activity of *Of*ChtII-C1C2 toward polymeric substrates was equal to that of *Of*Chi-h or *Of*ChtI. Therefore, the capacity for hydrolysis of crystalline chitin may be a function of the affinity of both the CAD and the CBM.

### Crystal structures of OfChtII-C1 and OfChtII-C2

**Figure 2.**
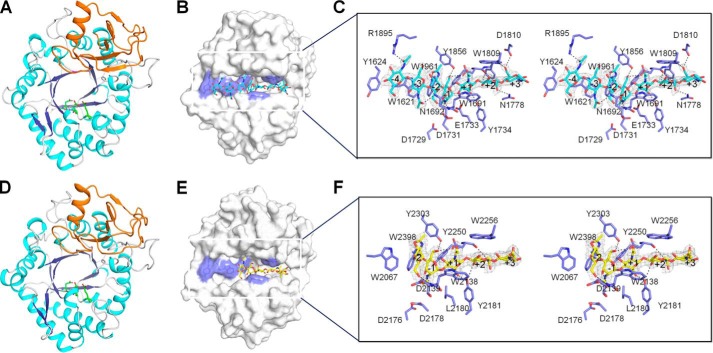
**Crystal structures of *Of*ChtII-C1 and *Of*ChtII-C2 in free form and in complex with oligosaccharide.** *A* and *D*, cartoon representation of *Of*ChtII-C1 (*A*) and *Of*ChtII-C2 (*D*). The structure consists of two domains: a core (β/α)_8_ TIM-barrel fold (*cyan*, α-helices; *blue*, β-strands) and an insertion domain (*orange*). The catalytic residues are shown as sticks with *green* carbon atoms. *B* and *E*, surface representations of *Of*ChtII-C1 complexed with (GlcNAc)_7_ (*B*) and *Of*ChtII-C2 E2180L complexed with (GlcNAc)_5_ (*E*). The ligands are shown as *sticks* with *cyan* and *yellow* carbon atoms, respectively. The aromatic residues that stack with the sugar rings are shown in *blue. C* and *F*, stereoview of the substrate-binding cleft with details of the interactions between (GlcNAc)_7_ and *Of*ChtII-C1 (*C*) and between (GlcNAc)_5_ and *Of*ChtII-C2 E2180L (*F*). The ligands are represented as sticks with *cyan* and *yellow* carbon atoms, respectively. The 2*F*_o_ − *F*_c_ electron-density map around the ligand is contoured at the 1.0 Å level. The catalytic residues and the amino acids that interact with the ligand are labeled and shown as *sticks* with *blue* carbon atoms. The *numbers* indicate the subsite to which the sugar is bound. Hydrogen bonds are drawn as *dashed lines*.

To illustrate the structural characteristics of OfChtII, *Of*ChtII-C1 and *Of*ChtII-C2 were crystallized and resolved. The crystal of *Of*ChtII-C1 was obtained by vapor diffusion, and the structure was determined using X-ray diffraction data at a resolution of 1.78 Å. The crystal belonged to space group P4_1_2_1_2, and each asymmetric unit contained a single molecule. According to the structural characteristics, *Of*ChtII-C1 could be divided into two distinct domains: a core domain and an insertion domain (CID) (Fig. 2). The core domain (residues 1613–1855 and 1940–1988, where the amino acid residues are numbered based on the full-length protein) is a classic (β/α)_8_ barrel with eight β-strands (β1–β8) tethered by eight α-helices (α1–α8). The CID (residues 1856–1939), which is composed of six antiparallel β-strands flanked by two short α-helices, is located between β7 and α7. The catalytic signature motif of GH18 chitinases, D*X*D*X*E (Asp^1729^–Glu^1733^), is located between β4 and α4. A long substrate-binding cleft, which comprises a series of conservative aromatic residues (Trp^1621^, Tyr^1624^, Trp^1663^, Trp^1691^, Trp^1809^, and Trp^1961^), is observed on the surface of *Of*ChtII-C1 (Fig. 2).

*Of*ChtII-C2 was also crystallized, and its crystal structure was resolved at a resolution of 1.95 Å. The P2_1_ asymmetric unit comprised two molecules of *Of*ChtII-C2 (which overlapped with an r.m.s. deviation of 0.24 Å over all atoms). *Of*ChtII-C2 showed 60% sequence identity with *Of*ChtII-C1. The overall architecture of *Of*ChtII-C2 was very similar to that of *Of*ChtII-C1, especially for the conserved (β/α)_8_ barrel domain (residues 2059–2301 and residues 2377–2426), corresponding to an r.m.s. deviation of 0.63 Å for 358 equivalent C^α^ atoms. However, subtle differences between *Of*ChtII-C1 and *Of*ChtII-C2 were observed. The entrance of the substrate-binding cleft in *Of*ChtII-C2 was more open than that in *Of*ChtII-C1. A conserved tryptophan on the surface of *Of*ChtII-C1 (Trp^1961^) was rotated 28.3° closer to the catalytic residue Glu^1733^ compared with the corresponding residue in *Of*ChtII-C2, which could make the substrate more accessible to the catalytic residues (Fig. S3).

### Substrate-binding cleft of OfChtII

To gain detailed insights into the substrate binding mode, catalysis defective mutants were constructed and crystallized. The inactive mutant, *Of*ChtII-C2 E2180L, enabled us to obtain the enzyme–substrate complex structure at a resolution of 2.4 Å by soaking the crystal with chitooligosaccharide substrate (GlcNAc)_5_ (Fig. 2, *D* and *E*)_._ The complex structure was similar to that of the wildtype enzyme, with an r.m.s. deviation of 0.17 Å based on the superimposition of 383 corresponding C^α^ atoms. The 2*F*_o_ − *F*_c_ map showed clear electron density for (GlcNAc)_5_ along the substrate-binding cleft from subsites −2 to +3. The sugar subsites are named according to Davies *et al.* (28), where subsite −*n* represents the non-reducing end, subsite +*n* represents the reducing end, and the enzymatic cleavage takes place between the −1 and the +1 subsites. A close-up view of the enzymatic cleavage region showed that the sugar residue bound at subsite −1 adopted an unfavorable “boat” ^1,4^*B* conformation, which raises the free energy of the substrate. The aromatic residues Trp^2067^, Trp^2138^, and Trp^2256^ interacted hydrophobically with the −2, +1, and +2 sugar residues, respectively. Additionally, all of the acetamido groups pointed away from their corresponding sugar rings, which suggests an energetically favorable conformation. In particular, the C2-acetamido group of the −1 sugar pointed toward the catalytic residue Asp^2178^, and the O1 and O6 atoms of the −1 sugar formed hydrogen bonds with the side chains of Tyr^2250^ and Tyr^2303^/Asp^2251^, respectively. The O6 atom of the −2 sugar formed hydrogen bonds with the main-chain amides of both Trp^2138^ and Asp^2139^. Trp^2181^ facilitated the binding of the +1 sugar by forming a hydrogen bond with O6 of the pyranose ring.

The inactive mutant of *Of*ChtII-C1 could not be crystallized. Therefore, the wildtype *Of*ChtII-C1 was incubated with chitooligosaccharides for a range of times. A structural snapshot was obtained in which a (GlcNAc)_7_ was observed (Fig. 2, *B* and *C*). The crystal structure was refined against 2.2 Å synchrotron data, yielding a final model with an *R* factor of 0.165 (*R*_free_ = 0.206; Table 2). The (GlcNAc)_7_ binds along the substrate-binding cleft and occupies subsites from −4 to +3, indicating the endo-acting activity of *Of*ChtII. In the electron density map, a partially cleaved glycosidic bond between sugar residues −1 and +1 was identified. The distance between C1 of −1 sugar and O4 of +1 sugar was 2.0 Å (C–O bond length is 1.4 Å, estimated standard error for bond length is 0.2 Å). The most significant of the enzyme–substrate contacts were localized in the area from subsites −2 to +2. Interactions between enzyme and substrate in this area, which were mediated by aromatic and charged residues, were similar to those observed in *Of*ChtII-C2 E2180L-(GlcNAc)_5_, except for the distorted sugar at subsite −1. In the *Of*ChtII-C1–substrate complex, the −1 sugar also adopted a boat ^1,4^*B* conformation, which was further stabilized by Tyr^1803^ and the catalytic residue Asp^1731^. In the substrate complex of the *Of*ChtII-C2 mutant, the corresponding aspartate turned to the other side as a result of the mutation of catalytic glutamate, which abolished the hydrogen bond. In addition, the −1 acetamido group in the *Of*ChtII-C1–substrate complex rotated around the C2-N2 bond toward the anomeric carbon, which is an energetically unfavorable conformation. The other sugar residues in (GlcNAc)_7_ made weaker interactions: the +3 sugar was stabilized by Tyr^1805^, Trp^1809^, and Asp^1811^; the −3 sugar stacked with Trp^1621^ and formed a hydrogen bond with Asn^1692^; and the −4 sugar stacked with Tyr^1624^ and formed a hydrogen bond with Arg^1895^.

**Table 2:** **X-ray data collection and structure-refinement statistics**

	*Of*ChII-C1	*Of*ChtII-C2	*Of*ChII-C1 + (GlcNAc)_7_	*Of*ChtII-C2 E2180L + (GlcNAc)_5_
Protein Data Bank entry	5Y29	5Y2A	5Y2B	5Y2C
Space group	P4_1_2_1_2	P2_1_	P4_1_2_1_2	P2_1_

**Unit-cell parameters**				
*a* (Å)	98.529	72.637	97.775	72.742
*b* (Å)	98.529	90.654	97.775	90.804
*c* (Å)	93.957	74.856	92.2331	74.625
α (°)	90.0	90.0	90.0	90.0
β (°)	90.0	116.4	90.0	116.3
γ (°)	90.0	90.0	90.0	90.0
Wavelength (Å)	0.97931	0.97776	0.97778	0.97776
Temperature (K)	100	100	100	100
Resolution (Å)	30.0–1.78 (1.81–1.78)	50.0–1.90 (1.93–1.90)	50.0–2.20 (2.24–2.20)	50.0–2.45 (2.49–2.45)
Unique reflections	43489	68407	23347	31828
Observed reflections	1,176,942	453,105	261,212	129,699
*R*_merge_	0.095 (0.496)	0.133 (0.486)	0.201 (0.967)	0.160 (0.446)
Average multiplicity	26.2 (26.9)	6.6 (5.8)	11.2 (8.4)	4.1 (3.0)
〈σ(*I*)〉	11.10 (10.18)	4.8 (3.04)	4.4 (2.83)	5.1 (2.87)
Completeness (%)	100 (100)	99.9 (99.9)	100 (100)	97.4 (95.5)
*R*/*R*_free_	0.1802/0.1985	0.1665/0.1859	0.1531/0.2022	0.1615/0.2030
Protein atoms	3023	6166	3023	6126
Water molecules	343	684	222	243
Other atoms	14	144	113	286

**R.m.s. deviation from ideal**				
Bond lengths (Å)	0.009	0.006	0.014	0.014
Bond angles (°)	0.88	0.99	1.33	1.52
Wilson B factor (Å^2^)	18.00	19.44	27.30	35.29
Average B factor (Å^2^)	22.88	21.50	29.42	35.62
Protein atoms	21.76	20.24	28.44	34.94
Water molecules	32.18	29.08	35.43	37.44
Ligand molecules			44.02	48.82

**Ramachandran plot (%)**				
Favored	98.4	98.3	97.9	97.6
Allowed	1.6	1.7	2.1	2.4
Outliers	0.0	0.0	0.0	0.0

## Discussion

### Comparison of OfChtI, OfChtII, and OfChi-h

Three chitinases, ChtI, ChtII, and Chi-h, are essential for molting in lepidopteran insects (29). However, the rationale for the requirement of such a complex mixture of enzymes has not been understood to date. Based on the structures of *Of*ChtII in this work and the structures of *Of*ChtI and *Of*Chi-h available from previous studies, a detailed structural comparison of the catalytic domains among *Of*ChtI, *Of*ChtII, and *Of*Chi-h revealed several differences in catalysis and substrate binding.

**Figure 3.**
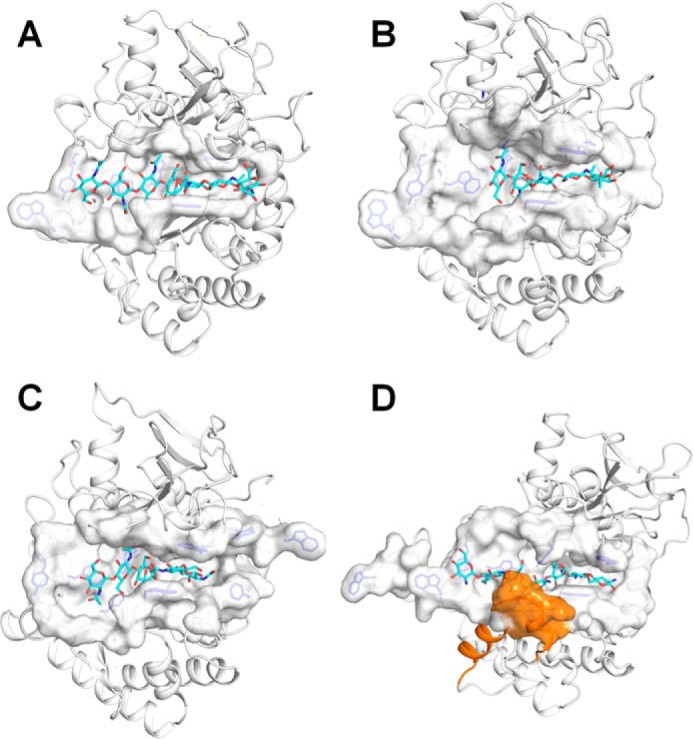
**Structural comparison of the substrate-binding cleft among three insect chitinases.** The substrate-binding clefts of *Of*ChtII-C1 (*A*), *Of*ChtII-C2 (*B*), *OfChtI* (*C*), and *Of*Chi-h (*D*) are shown as surface, and the rest of the regions of the 1enzymes are shown as cartoons. The (GlcNAc)_7_ bound to *Of*ChtII-C1, the (GlcNAc)_5_ bound to *Of*ChtII-C2, the (GlcNAc)_2/3_ bound to *Of*ChtI and the (GlcN)_7_ bound to *Of*Chi-h are shown as *sticks* with *cyan* carbon atoms. The aromatic residues in the substrate-binding cleft are shown as *blue sticks*. In *Of*Chi-h, the unique structural element comprises of two helices is colored *orange*.

First, although all of these enzymes possess a long tunnel-like cleft with both ends open, the distribution of aromatic residues and subsite occupancy along the substrate-binding cleft showed subtle differences (Fig. 3). Minor differences in the substrate-binding cleft could confer different hydrolysis patterns of chitinases. For *Of*ChtII, although the distribution of aromatic residues along the substrate-binding cleft was asymmetric, the subsite occupancy was symmetrical because the most significant interactions were localized in a symmetrical area from sugar subsites −2 to +2. In *Of*ChtI, which is regarded as an endo-acting chitinase, 10 aromatic residues are distributed symmetrically around the catalytic center, and subsite occupancy is also symmetrical. Furthermore, the structure of the enzyme–substrate complex indicates that (GlcNAc)_6_ may occupy the substrate-binding cleft symmetrically (10). For *Of*Chi-h, which exhibits the characteristics of an exo-acting chitinase, the distribution of aromatic residues around active sites is asymmetric. The structure of *Of*Chi-h complexed with chitoheptaose ((GlcN)_7_, a substrate mimic) shows that (GlcN)_7_ occupies the subsites from −5 to +2. Together, these observations indicate that the catalytic domains of *Of*ChtII possess substrate-binding clefts with structural characteristics similar to those found in both *Of*Chi-h and *Of*ChtI.

Second, in *Of*ChtII and in *Of*ChtI, two short β sheets are present in the wall of the substrate-binding cleft, forming an open, flat tunnel. However, in *Of*Chi-h, two extra α-helices formed by a unique sequence are observed at the same position, which deepens and narrows the cleft (Fig. 3*D*). This structural element is speculated to increase its affinity for chitin chains.

Third, the structural components for binding of crystalline chitin differ among these enzymes. *Of*ChtI possesses a hydrophobic plane formed by four highly conserved aromatic residues that are important for binding crystalline chitin (10). *Of*Chi-h has a deep, narrow binding cleft that is suitable for binding single chitin chains rather than crystalline chitin. In the two catalytic domains of *Of*ChtII, there were no significant structural characteristics that would contributing to binding of crystalline chitin. Nevertheless, given the large number of CBMs, whose ability for the binding of polymeric chitin has been confirmed in this and other studies (30–33), it is reasonable to believe that the full-length ChtII may have a high potential for hydrolyzing crystalline chitin. Although the efforts to obtain the full-length *Of*ChtII fell through, the high hydrolytic activity and binding affinity of *Of*ChtII-B4C1 toward polymeric chitins provide forceful evidences of the high efficiency of full-length enzyme (Fig. 1*B* and Fig. S4).

### Comparison of substrate–enzyme interactions

Chitooligosaccharide substrates binding to chitinases mainly rely on π–π stacking and/or hydrophobic interactions between the sugar and aromatic residues. The aromatic residues near the catalytic center are conserved, especially in the subsites −3 to +2. Comparison between the complex structures of enzymes, *Of*ChtIII-C1, *Of*ChtIII-C2, *Of*ChtI, and *Of*Chi-h, indicates that four tryptophans, Trp^1621^, Trp^1961^, Trp^1691^, and Trp^1809^ (*Of*ChtII-C1) are highly conserved. They form stacking interactions with five sugars (−3 to +2). Other conserved interactions include hydrogen bonds between the C3/C6-hydroxyl group of −1 sugar with the enzyme. However, interactions beyond this area (−3 to +2) is different. In *Of*ChtI, a phenylalanine (Phe^194^) well stacks with +3 sugar. However, this aromatic residue is substituted by Met^1780^/Arg^2227^ in *Of*ChtII, in which the +3 sugar is stabilized by hydrogen bonds. In *Of*Chi-h, +3 subsite is absent.

### Comparison of chitinase systems between S. marecesens and O. furnacalis

Relative to the extensively studied *S. marcescens*, the chitinolytic system of *O. furnacalis* also involves three chitinases, but they may work in a different manner. Insect Chi-h is presumed to be obtained from bacteria by gene horizontal transfer (34, 35). The sequence and structure of *Of*Chi-h are remarkably similar to those of *Sm*ChiA (73% sequence identity with an r.m.s. deviation of 1.3 Å for 534 C^α^ atoms). Previous report showed that *Of*Chi-h and *Sm*ChiA also exhibited similar substrate-binding sites and hydrolytic anomeric products composition (11). The active site architecture of *Of*ChtI is reminiscent to that of *Sm*ChiB, because they both prefer to act from the non-reducing end. However, the exo-acting *Sm*ChiB has a blocked and tunnel-like substrate-binding cleft, but *Of*ChtI possesses an open and symmetric cleft (10, 36). *Of*ChtII is unique. Its two endo-acting catalytic domains, *Of*ChtII-C1 and *Of*ChtII-C2, are structurally identical. Structural superimposition of *Of*ChtII-C1 with *Sm*ChiC indicates an r.m.s. deviation of 3.94 Å for 266 equivalent C^α^ atoms. *Sm*ChiC lacks the α+β insertion domain, which is responsible for forming one “wall” of the substrate-binding cleft of *Of*ChtII-C1. Moreover, *Of*ChtII possesses a long and deep substrate-binding cleft lined with 12 aromatic residues, whereas *Sm*ChiC has a much shallower substrate-binding cleft composed of 8 aromatic residues. Although both *Of*ChtII-C1 and *Sm*ChiC are endo-chitinases, these structural differences accounts for a big difference in catalytic efficiency. We recently succeeded in obtaining a truncated *Of*ChtII-B4C1 that contains three adjacent CBMs followed by one catalytic domain C1. By using α-chitin as substrate, *Of*ChtII-B4C1 exhibited as high as 1.7 times activity than *Sm*ChiC (Fig. S5). Taken together, we conclude that the insect *O. furnacalis* has a different chitinase system from the bacterium *S. marecesens*.

Taken together, the structural and biochemical characteristics of *Of*ChtII presented here provide important insights into the chitin-degrading system of insects. Three chitinases, ChtI, ChtII, and Chi-h, utilize different hydrolysis patterns for effective turnover of chitin. Being up-regulated 1–2 days before molting,^4^ *Of*ChtII may act as the first enzyme in chitin degradation. The multiple CBMs and inactive catalytic domains in the N terminus may serve as anchors during the binding of enzyme and substrate on the outermost exposed surface of chitin microfibers, which probably exhibit high affinity to crystalline chitin and low off-rates. The two active catalytic domains and other domains are linked with highly flexible proline-rich hinge regions, resulting in a random and alterable positioning of C1 and C2 and adjacent chains. Therefore, *Of*ChtII could ablate the surface of chitin fibrils and break crystalline chitin into small pieces, which then become accessible to *Of*ChtI and *Of*Chi-h. Chi-h is an exo-acting processive chitinase that degrades chitin chains from their reducing ends (11), whereas *Of*ChtI acts as an endo-processive chitinase and works with *Of*Chi-h by a synergistic mechanism (10, 36). This work also offers a crucial starting point for designing agrochemicals targeting molting of insect pests.

## Experimental procedures

### Gene cloning and site-directed mutagenesis

Total RNAs were extracted from the pupae of *O. furnacalis* and subjected to reverse transcription using PrimeScript^TM^ RT reagent kit (TaKaRa) according to the manufacturer's instructions. The resulting cDNA was used as a template to amplify the full-length nucleotide sequence of *Of*ChtII.

Gene-specific primers for *Of*ChtII were designed and synthesized according to the highly conserved amino acid sequences based on the multiple sequence alignment of other known insect chitinases. A 252-bp fragment (D1) was nested PCR (first PCR, D1-F-outer, D1-R-outer; second PCR, D1-F-inner, D1-R-inner) amplified from cDNA. The 3′ ends of the transcript were then obtained by performing 3′ RACE twice (3-1, amplified by first 3′ RACE, 3R1-outer, 3R1-inner; 3–2, amplified by second 3′ RACE, 3R2-outer, 3R2-inner). The 5′ sequences of the transcript (5-1) were amplified by 5′ RACE (5R1-outer, 5R1-inner). According to the known sequences and conserved sequences, another fragment (D2) was used in a nested PCR (first PCR, D2-F-outer, D2-R-outer; second PCR, D2-F-inner, D2-R-inner) amplified. The 5′ ends of *OfChtII* were then obtained five times SMARTer 5′ RACE (TaKaRa). 5-2, amplified by 5R2-outer, 5R2-inner; 5-3, amplified by 5R3-outer, 5R3-inner; 5-4, amplified by 5R4-outer, 5R4-inner; 5-5, amplified by 5R5-outer, 5R5-inner; and 5-6, amplified by 5R6-outer, 5R6-inner). All of these sequences were spliced and verified by PCR. The overall strategy is summarized in Fig. S6, and the sequences of the primers are listed in Table S1.

The expression constructs for *Of*ChtII-C1 or *Of*ChtII-C2 were synthesized after codon optimization for yeast expression (Taihe Biotechnology Co.). The mutants were produced by the QuikChange site-directed mutagenesis kit (Stratagene).

### Protein expression and purification

The recombinant plasmids containing *Of*ChtII-C1, *Of*ChtII-C2, *Of*ChtII-C1C2, or *Of*ChtII-B4C1 gene were transformed into *Pichia pastoris* GS115 strain (Invitrogen) using the electroporation-mediated method. The positive clones carrying His^+^ and Mut^+^ traits were selected using histidine auxotroph medium and verified by PCR. The positive clone was first grown in 200 ml of buffered glycerol complex medium (1% yeast extract, 1% glycerol, 2% peptone, 0.2% biotin, 1.34% yeast nitrogen, 100 mm potassium phosphate, pH 6.0) at 30 °C. When the *A*_600_ reached 2.0, the cells were collected and resuspended in 1 liter of buffered methanol complex medium (1% yeast extract, 1% methanol, 2% peptone, 0.2% biotin, 1.34% yeast nitrogen, and 100 mm potassium phosphate, pH 6.0). Methanol was added to a final concentration of 1% (v/v) every day. The culture supernatant was harvested by centrifugation 6000 × *g* for 10 min after 120 h of fermentation and subjected to ammonium sulfate precipitation (75% saturation) at 4 °C.

The precipitate was resuspended in distilled water and then desalted in buffer A (20 mm sodium phosphate, 0.5 m sodium chloride, pH 7.4). The resuspension solution was centrifuged at 17,000 × *g* for 30 min at 4 °C and passed through a 0.2-μm filter. Afterward, the sample was loaded onto a 5-ml HisTrap FF affinity column (GE Healthcare) pre-equilibrated with buffer A. To remove non-specifically bound proteins, the column was first washed with buffer A containing 20 mm imidazole and then with buffer A containing 50 mm imidazole. Finally, the target protein (*Of*ChtII-C1, *Of*ChtII-C2, *Of*ChtII-C1C2, or *Of*ChtII-B4C1) was eluted with buffer B (20 mm sodium phosphate, 0.5 m sodium chloride, 250 mm imidazole, pH 7.4). The protein concentration was measured using a Bradford protein assay kit with bovine serum albumin as a standard protein, and the purity of the sample was analyzed by SDS-PAGE. Mutants were expressed and purified using the same procedure. *Of*ChtI, *Of*ChtI-CAD, and *Of*Chi-h were expressed and purified as previously described (10, 11). The yields for the recombinant proteins are ∼12 mg/liter (*Of*ChtII-C1), 15 mg/liter (*Of*ChtII-C1), 10 mg/liter (*Of*ChtII-C1C2), 1 mg/liter (*Of*ChtII-B4C1), 15 mg/liter (*Of*ChtI-CAD), 3 mg/liter (*Of*ChtI), and 20 mg/liter (*Of*Chi-h).

### Enzymatic activity assays

Four kinds of substrates were used for chitinase activity assays, including MU-(GlcNAc)_2_ (Sigma–Aldrich), colloidal chitin, α-chitin (Sigma–Aldrich) and β-chitin (a kindly gift from Prof. Yuguang Du from Institute of Process Engineering, Chinese Academy of Science). For MU-(GlcNAc)_2_, Michaelis–Menten parameters were determined. Reaction components were incubated in a final volume of 100 μl at 303 K for 20 min in the presence of 20 mm sodium phosphate (pH 6.0), enzyme (*Of*ChtII-C1, 11.6 nm; *Of*ChtII-C2, 23.8 nm; *Of*ChtII-C1C2, 6.7 nm; *Of*ChtII-B4C1, 10.0 nm; *Of*Chi-h, 1.0 nm, *Of*ChtI-CAD, 1.0 nm; *Of*ChtI, 2.0 nm), and 0.25–50 μm MU-(GlcNAc)_2_. Then enzyme reaction was stopped by the addition of 100 μl of 0.5 m sodium carbonate solution, and fluorescence of the released 4-methylumbelliferone was quantified (excitation, 366 nm; emission, 445 nm). Data analysis was performed with OriginPro 8.5 (OriginLab). For the three polymeric substrates, the activities were assayed in time-course experiments. Reaction mixtures contained enzyme as indicated (1.0 μm for colloidal chitin; 2.0 μm for α-chitin and β-chitin) and 3 mg/ml substrate, 20 mm sodium phosphate buffer (pH 6.0) to a final volume of 100 μl. After incubating at 30 °C for an appropriate time, the sample was centrifuged at 12,000 × *g* for 10 min. 60 μl of supernatant was removed, and the amount of reducing sugars was determined by the potassium ferricyanide method (37).

### Chitin-binding assays

α-Chitin was used as the substrate for chitin binding assays. The reaction mixtures contained 10 mg/ml α-chitin, 0.2 mg/ml enzyme, 150 mm sodium chloride, and 20 mm sodium phosphate buffer (pH 6.0). Bovine serum albumin was used as a negative control. The reaction mixtures were incubated at 4 °C with rotation. At different time points, the samples were centrifuged at 6000 × *g* for 5 min, and the protein concentrations were determined by Bradford assays.

### Crystallization and structure determination

Because *Of*ChtII-C1C2 was unstable, the linker between two catalytic active domains was autocleaved during crystallization; only the crystals of individual CAD, *Of*ChtII-C1, and *Of*ChtII-C2, were obtained. The extra amino acids in the CID of ChtII-C1 seriously influenced its expression. Thus, residues 1863–1878 (GDKWDSPREQWRKDAN; Fig. S1) were replaced by ENRGIH, the corresponding residues in ChtII of *Bombyx mori*. Pure protein was desalted in 20 mm Tris-HCl (pH 7.5), 50 mm NaCl and spin-concentrated to 10.0 mg/ml. Hanging-drop vapor-diffusion crystallization experiments were set up at 277 K by mixing 1 μl of reservoir solution and 1 μl of sample. Crystallization screening of recombinant *Of*ChtII-C1 and *Of*ChtII-C2 was performed using the commercially available JCSG Core Suites I-IV (Qiagen) as well as Index, Crystal Screen and Crystal Screen 2 (Hampton Research, Riverside, CA) kits. *Of*ChtII-C1 crystals appeared in Index 73 (0.2 m sodium chloride, 0.1 m Tris, pH 8.5, and 25% PEG3350), whereas *Of*ChtII-C2 crystals appeared in Index 51 (0.2 m ammonium acetate, 0.1 m Bis-Tris, pH 6.5, and 45% 2-methyl-2,4-pentadiol). The mutant, *Of*ChtII-C2 E2180L, crystallized in the same condition.

The crystals were cryoprotected by a 5-s immersion in a solution containing reservoir solution and 25% (v/v) glycerol and subsequently flash-cooled in liquid nitrogen. For the enzyme–substrate complex structure, the crystal was soaked in a drop containing reservoir solution and chitooligosaccharides for 5–30 min before cryoprotection. The diffraction data were collected on BL18U at the Shanghai Synchrotron Radiation Facility in China, and the diffraction data were processed using the HKL-3000 package (38).

The structures of native *Of*ChtII-C1 and *Of*ChtII-C2 were solved by molecular replacement with Phaser (39) using the structure of human chitotriosidase (Protein Data Bank entry 1GUV) as a model. The subsequent complex structures were solved using the coordinates of free proteins as models. Structure refinement was performed by PHENIX suite of programs (40). The molecular models were manually built and extended using Coot (41). The structural figures were generated using PyMOL (DeLano Scientific, San Carlos, CA). The data-collection and structure-refinement statistics are summarized in Table 2.

## Author contributions

W. C. and M. Q. investigation; W. C. methodology; W. C. writing-original draft; M. Q. resources; M. Q. supervision; Y. Z. data curation; Y. Z. software; Q. Y. conceptualization; Q. Y. funding acquisition; Q. Y. writing-review and editing.

## Supplementary Material

Supporting Information

## Footnotes

4 M. Qu and Q. Yang, unpublished results.

## Footnotes

3 The abbreviations used are:

GH: glycoside hydrolase family
ChtII: insect group II chitinase
ChtI: insect group I chitinase
Chi-h: chitinase-h
*Sm*ChiA: chitinase A from *S. marecesens*
*Sm*ChiB: chitinase B from *S. marecesens*
*Sm*ChiC: chitinase C from *S. marecesens*
*Of*ChtI: ChtI from *O. furnacalis*
*Of*ChtII: ChtII from *O. furnacalis*
*Of*Chi-h: Chi-h from *O. furnacalis*
CAD: chitinase-like domain
CBM: chitin-binding module
MU-(GlcNAc)_2_: 4-methylumbelliferyl β-d-*N*,*N′*-diacetylchitobioside hydrate
CID: chitinase insertion domain
r.m.s.: root mean square
cDNA: complementary DNA
RACE: rapid amplification of cDNA ends.
