# Supplementary material for: Structural analysis of group II chitinase (ChtII) catalysis completes the puzzle of chitin hydrolysis in insects
Source: J Biol Chem. 2018 Jan 9;293(8):2652–60. doi: 10.1074/jbc.RA117.000119 (PMC5827449; doi:10.1074/jbc.RA117.000119)
Supplement: Supporting Information [file supp_293_8_2652__index.html]

Structural analysis of group II chitinase (ChtII) catalysis completes the puzzle of chitin hydrolysis in insects — Structural analysis of insect group II chitinase — Structural analysis of group II chitinase (ChtII) catalysis completes the puzzle of chitin hydrolysis in insects — Structural analysis of insect group II chitinase — Supporting Information 

# Structural analysis of group II chitinase (ChtII) catalysis completes the puzzle of chitin hydrolysis in insects

## Supporting Information

- Supporting Information - Supporting Information
