# Supplementary material for: Structural analysis of group II chitinase (ChtII) catalysis completes the puzzle of chitin hydrolysis in insects
Source: J Biol Chem. 2018 Jan 9;293(8):2652–60. doi: 10.1074/jbc.RA117.000119 (PMC5827449; doi:10.1074/jbc.RA117.000119)
Supplement: Supporting Information [file supp_RA117.000119_132660_2_supp_48905_p22lkz.pdf]

SUPPLEMENTAL DATA

Structural analysis of group II chitinase (ChtII) catalysis completes the puzzle of chitin hydrolysis in insects

Wei Chen[1,#], Mingbo Qu[1,#], Yong Zhou[1], Qing Yang[1,2,*]

[1]State Key Laboratory of Fine Chemical Engineering, School of Life Science and Biotechnology and School of Software, Dalian University of Technology, 2 Linggong Road, Dalian 116024, China;

[2]Institute of Plant Protection, Chinese Academy of Agricultural Sciences, 2 West Yuanmingyuan Road, Beijing 100193, China.

Supplementary Table S1
Supplementary Figure S1
Supplementary Figure S2
Supplementary Figure S3
Supplementary Figure S4
Supplementary Figure S5
Supplementary Figure S6

Table S1. Primers used during cloning and RT-PCR

**PCR fragment/ Conserved sequence**	**Primer**	**Sequence (5'-3')**
D1	D1-F-outer	CTTGGGCGATAGACTTGGACGAC
	D1-R-outer	GGRTACTCCCAGTSGAKGTC
	D1-F-inner	TGTGAGAAGTTCTACGTTTGCGTGA
	D1-R-inner	CCAGTSGAKGTCCAKYCCGTCRAA
3-1	3R1-outer	TGCAACTCAACGATGAAGTTCCT
	3R1-inner	GTACGCAATGCTCCCGACTGACT
3-2	3R2-outer	AGTGCCAGTTTTAGCTCGCCTTCT
	3R2-inner	CCAACGGCCTTAATATTCCTGTC
5-1	5R1-outer	AGACGGGTTTTGTAGGCTTAGAGG
	5R1-inner	GAGTTGCATAGAGGTAGATTCCC
D2	D2-F-outer	GGAMHGACTCVSHAGGVGAYAARTA
	D2-R-outer	TAGTGGTGGTCGTGCTGGGCGT
	D2-F-inner	TTGGAACTATCCCGTTTGCTGGC
	D2-R-inner	CACAGCAGCGGTTGTTGAAGTC
5-2	5R2-outer	CGCCACCTTTTAGAAGAGCTTTGAT
	5R2-inner	CAGGAACAAGTGGGGTATGATGCC
5-3	5R3-outer	CATAATTCGCCTTATCGGACGCTG
	5R3-inner	GAAGTTGTGCATGCGCAGGAAAGGTA
5-4	5R4-outer	CCCAATCCAAGGAGTACTTTGACG
	5R4-inner	GTCGGTCCTCTTCGCACAGCATT
5-5	5R5-outer	ATACACTTAGCGACATCCAAATGACC
	5R5-inner	CGTTGAAATGGTGCTCAGTATTGGGA
5-6	5R6-outer	GTCGAATAATGCGAATGAATTGCCAG
	5R6-inner	TGCCAGCTTCATCGAATGCAACCAC
ChtII full length	ChtII-F-outer	TCGCGACACGCACGGCCTAAACGCA
	ChtII-R-outer	ACGGCCTAAACGCAGGCGCATCTTC
	ChtII-F-inner	GCAAGTACCTATATTGAACG
	ChtII-R-inner	GCTAGCGTGGCTATTCCTAGTCTAA

Supplementary Figure

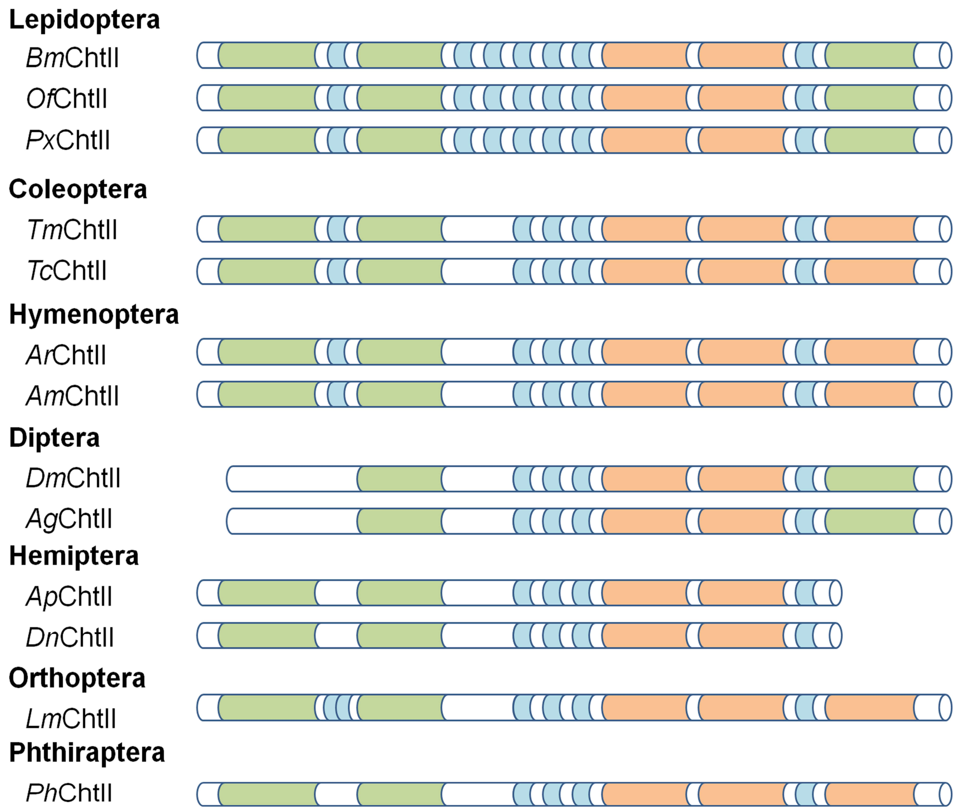

Figure S1. Domain organization of group II chitinases in different insect orders. Active catalytic domains, inactive catalytic domains, chitin binding domains and linker regions are highlighted in orange, green, blue and white, respectively. Group II chitinases compared are: *Bm*ChtII, *Bombyx mori* (BGIBMGA006874-TA); *Px*ChtII, *Plutella xylostella* (XP_001655973.1); *Tm*ChtII, *Tenebrio molitor* (CAD31740.4); *Tc*ChtII, *Tribolium castaneum* (NP_001036067.1); *Ar*ChtII, *Athalia rosae* (XP_012269359.1); *Am*ChtII, *Apis mellifera* (XP_006570346.1); *Dm*ChtII, *Drosophila melanogaster* (NP_001036422.1); *Ag*ChtII, *Anopheles gambiae* (XP_001238192.2); *Ap*ChtII, *Acyrthosiphon pisum* (XP_001943038.1); *Dn*ChtII, *Diuraphis noxia* (XP_015370321.1); *Lm*ChtII, *Locusta migratoria* (AMT75074.1); *Ph*ChtII, *Pediculus humanus corporis* (XP_002426510.1).

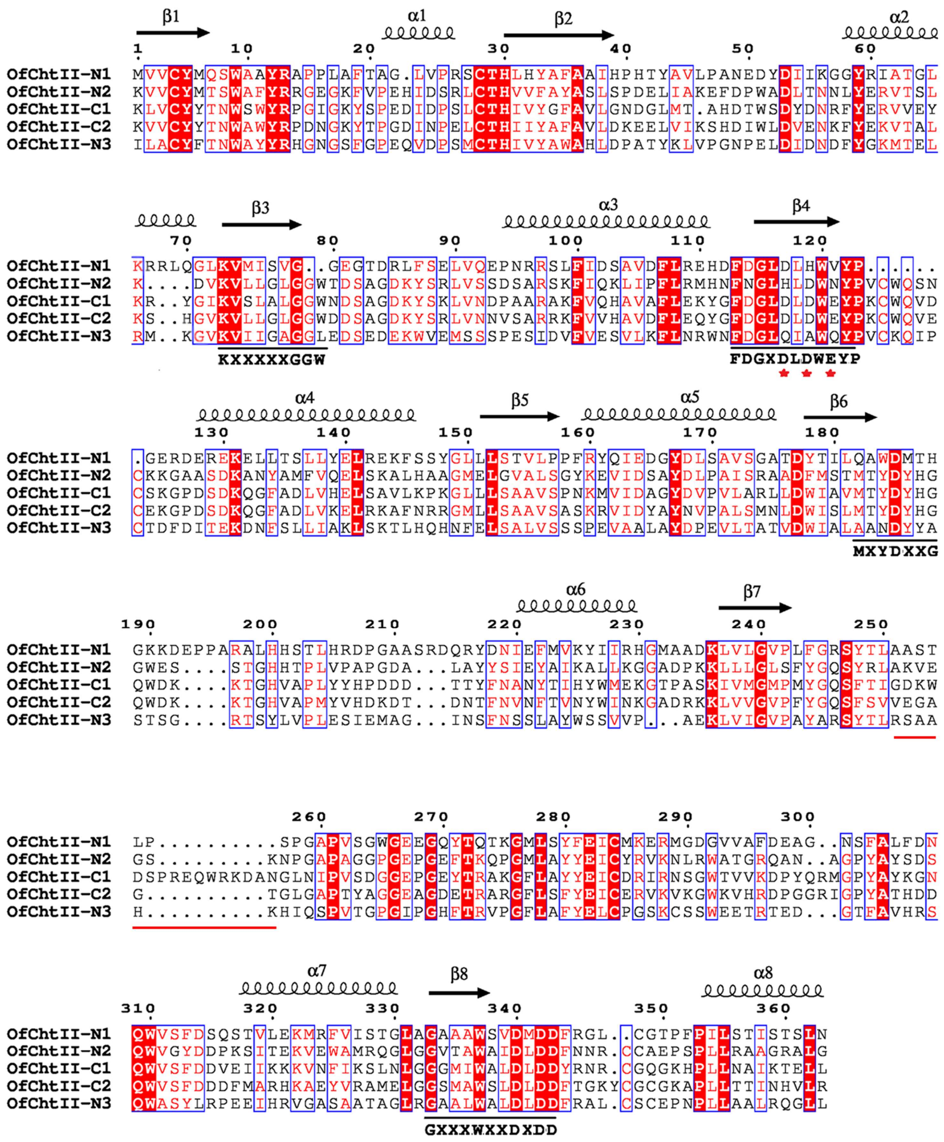

Figure S2. Multiple sequence alignment of *Of*ChtII catalytic domains. Sequence alignment of five deduced catalytic domains of *Of*ChtII, made with ESPript. The secondary structure of the core TIM barrel and the highly conserved motifs are indicated. Catalytic residues are indicated by asterisk. The residues replaced during *Of*ChtII-C1 crystallization are indicated by red line.

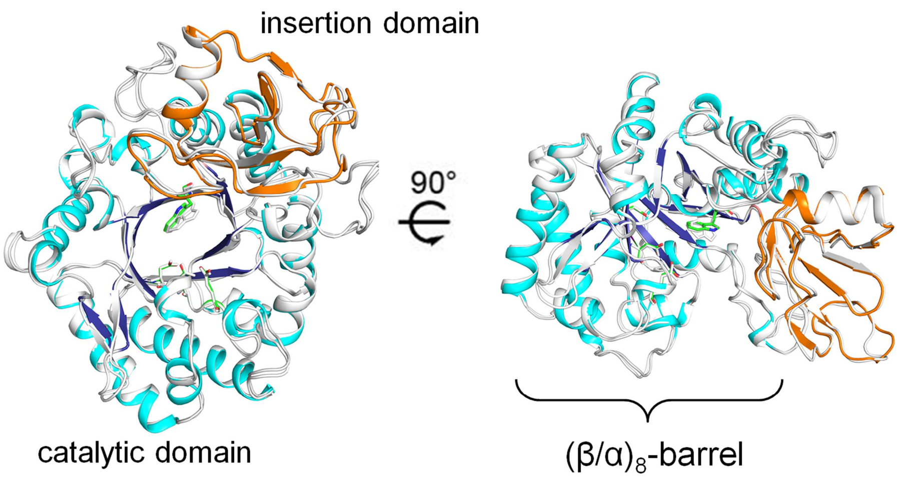

Figure S3. Structural comparison of *Of*ChtII-C1 and *Of*ChtII-C2.

Two perpendicular views of superimposed *Of*ChtII-C1 and *Of*ChtII-C2 reveal that the catalytic domains are folded into an identical $(\beta/\alpha)_8$-barrel, while the insertion domains display a slight variation. *Of*ChtII-C2 is colored white; the α-helices and β-strands of catalytic domain for *Of*ChtII-C1 are colored cyan and blue, respectively. The insertion domain of *Of*ChtII-C1 are colored orange. The catalytic residues and Trp^{1961} of *Of*ChtII-C1 are shown as sticks with green carbon atoms.

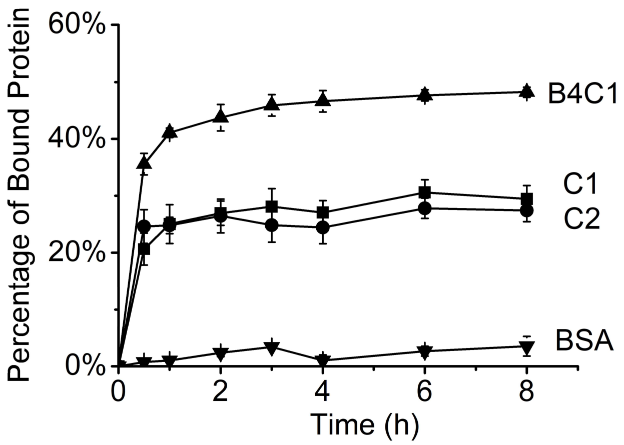

Figure S4. The binding affinity of *Of*ChtII truncations. The concentration of free protein in the supernatant was determined at different time points after incubation with α-chitin. The results are the average of three independent repeats, with the standard deviations indicated.

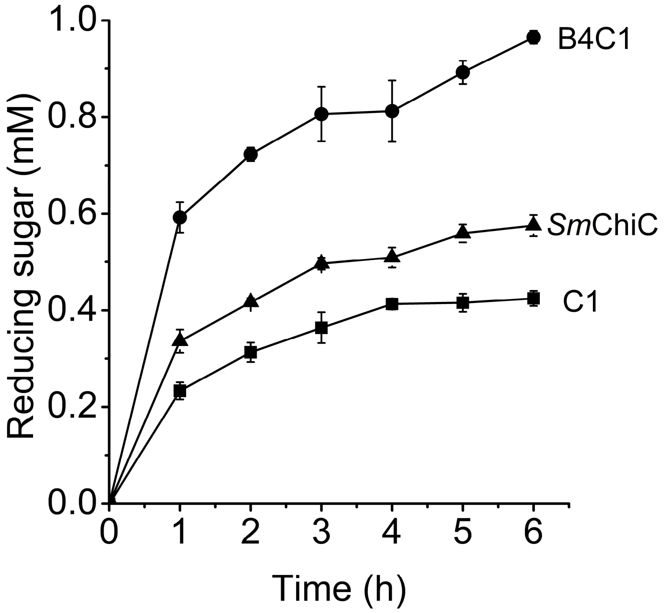

Figure S5. The comparison of hydrolysis activity towards α-chitin between *Sm*ChiC and *Of*ChtII truncates.

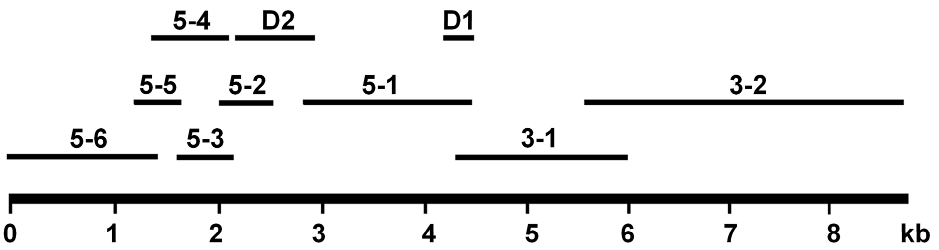

Figure S6. The overall strategy used in the gene cloning of *Of*ChtII
